# Safety and efficacy of edaravone in patients with amyotrophic lateral sclerosis: a systematic review and meta-analysis

**DOI:** 10.1007/s10072-023-06869-8

**Published:** 2023-05-30

**Authors:** Anas Zakarya Nourelden, Ibrahim Kamal, Abdulrahman Ibrahim Hagrass, Abdelrahman G. Tawfik, Mahmoud M. Elhady, Ahmed Hashem Fathallah, Mona Muhe Eldeen Eshag, Mohamed Sayed Zaazouee

**Affiliations:** 1 Faculty of Medicine, Al-Azhar University, Cairo, Egypt; 2 Department of Pharmacotherapy, College of Pharmacy, The University of Utah, Salt Lake City, UT USA; 3 Faculty of Medicine, Benha University, Qalubiya, Egypt; 4 Faculty of Medicine, Minia University, Minia, Egypt; 5 Faculty of Medicine, University of Bahri, Khartoum, Sudan; 6 Faculty of Medicine, Al-Azhar University, Assiut, Egypt

**Keywords:** Edaravone, ALS, Amyotrophic lateral sclerosis, Meta-analysis

## Abstract

**Aim:**

The study aims to increase understanding of edaravone’s efficacy and safety as an amyotrophic lateral sclerosis (ALS) treatment and provide significant insights regarding this field’s future research.

**Methods:**

We conducted a comprehensive search of the Embase, PubMed, Cochrane Library, Web of Science, and Scopus databases for randomized controlled trials and observational studies up until September 2022. We evaluated the studies’ quality using the Cochrane risk of bias tool and the National Institutes of Health tool.

**Results:**

We included 11 studies with 2845 ALS patients. We found that edaravone improved the survival rate at 18, 24, and 30 months (risk ratio (RR) = 1.03, 95% confidence interval (CI) [1.02 to 1.24], *P* = 0.02), (RR = 1.22, 95% CI [1.06 to 1.41], *P* = 0.007), and (RR = 1.17, 95% CI [1.01 to 1.34], *P* = 0.03), respectively. However, the administration of edaravone did not result in any significant difference in adverse effects or efficacy outcomes between the two groups, as indicated by a *P* value greater than 0.05.

**Conclusion:**

Edaravone improves survival rates of ALS patients at 18, 24, and 30 months with no adverse effects. However, edaravone does not affect functional outcomes. In order to ensure the validity of our findings and assess the results in accordance with the disease stage, it is essential to carry out additional prospective, rigorous, and high-quality clinical trials. The current study offers preliminary indications regarding the effectiveness and safety of edaravone. However, further comprehensive research is required to establish the generalizability and sustainability of the findings.

**Supplementary Information:**

The online version contains supplementary material available at 10.1007/s10072-023-06869-8.

## Introduction

Amyotrophic lateral sclerosis (ALS) is a degenerative neurological condition characterized by the gradual deterioration of the motor neurons located in the brain and spinal cord, which are responsible for regulating voluntary muscle movement [1, 2]. ALS is distinguished by muscular weakness, atrophy, and spasticity, resulting in challenges with speech, swallowing, and respiration. It is estimated that there are 4.42 cases of ALS for every 100,000 people in the world’s population, whereas there are 7.7 cases of ALS for every 100,000 people in the USA [3, 4]. Unfortunately, most patients die within 2–3 years after diagnosis due to the failure of respiratory muscles, with a median survival time ranging from 1.5 to 4 years [5, 6]. Although 90 – 95% of cases are sporadic, 5–10% of patients are considered to have the familial form of ALS [5]. It increases the economic burden on health systems [7]. The national cost of ALS may reach € 130 million [7].

Although the pathophysiological mechanisms and environmental factors that affect the disease are still unclear, the evidence suggests that free radicals play a crucial role in the progression of ALS [8, 9]. Free radicals can harm the central nervous system (CNS) by limited ability to scavenge free radicals and weak capability for regeneration [10]. Furthermore, reactive oxygen species (ROS) cause genetic mutations by changing the actions of some enzymes, such as superoxide dismutase and glutamate synthetase [11]. The literature supports the oxidative stress hypothesis by concluding that the CNS tissue of ALS patients contained a higher level of 3-nitrotyrosine (3-NT), a biomarker for oxidative stress [12].

Although many drugs have been tested to treat ALS, riluzole was the only approved drug for about 20 years. Riluzole functions as an anti-glutamatergic agent by diminishing the discharge of glutamate, a neurotransmitter that is hypothesized to be involved in the deterioration of motor neurons in ALS. Clinical trials have demonstrated that riluzole has the potential to decelerate the advancement of ALS and extend the patient’s lifespan [13, 14]. Additional drugs, such as Nuedexta, have received authorization for the management of ALS. It is hypothesized that this drug selectively addresses distinct manifestations of ALS, specifically emotional instability.

Edaravone is a newly tested free radical-scavenging medication that prevents neural cell damage by eliminating hydroxyl radicals and lipid peroxides [15, 16]. Many studies investigated the antioxidant effects of edaravone [17–21]. Yoshino and Kimura concluded that following 14 days of edaravone treatment, the level of 3NT in CSF fluid was considerably lower than at the beginning [22]. Also, edaravone was found to increase plasma uric acid, indicating that it effectively scavenges peroxynitrite [23]. This study aims to update the evidence of edaravone regarding efficacy, safety, and survival outcomes in ALS patients.

## Methods

The authors conducted a systematic review and meta-analysis in adherence to the most recent PRISMA updates and Cochrane standards [24, 25].

### Literature search and data collection

We searched PubMed, Cochrane library, Embase, Scopus, and Web of Science databases for relevant trials until September 2022. Our search was done using the following search strategy: (edaravone OR norantipyrine OR norphenazone OR edarabone OR radicava OR fraseda OR radicut OR phenylmethylpyrazolone OR methylphenylpyrazolone OR nuravon OR aravon OR “MCI 186” OR “MCI-186” OR “MCI186”) AND (“amyotrophic lateral sclerosis” OR ALS OR “gehrig’s disease” OR “gehrig disease” OR “gehrigs disease” OR “lou gehrig’s” OR “lou-gehrigs” OR “lou gehrig disease”).

### Studies selection and eligibility criteria

We included randomized control trials (RCTs) and observational studies that compared edaravone versus control in patients with ALS regarding safety, efficacy, and survival outcomes. We removed the duplicates using EndNote software and screened the title and abstract of all the remaining studies. Studies that might fit the eligibility criteria were full-text screened. Additionally, we screened the references of the included articles to find any missing relevant studies. Two authors did the previous steps, and the third author solved any conflict.

### Quality assessment

The Cochrane risk of bias tool (version 1) was used to assess the degree to which the studies under consideration may have been biased [24]. The purpose of this tool is to evaluate diverse forms of bias that may potentially impact the reliability of the study’s outcomes. The application of this tool offers a systematic and precise methodology for identifying and addressing potential bias factors, thus enhancing the general quality and dependability of the research outcomes. Non-RCTs were assessed by the NIH tool [26]. NIH tool for observational studies is composed of 14 questions, and the details about each question are provided in this reference [26]. The quality of evidence for the analyzed outcomes was evaluated using the GRADE methodology (version 20 of GRADEpro, McMaster University, 2013) [27].

### Data extraction

The following data were retrieved in Excel sheets:

1. Summary data: study design, site, registration, follow-up duration, inclusion criteria, and outcomes.
2. Baseline data: arms, patients’ number, age, gender, ALS diagnosis definite ALS diagnosis probable, ALS severity grade, duration of disease, riluzole use, and ALS Functional Rating Scale-Revised (ALSFRS-R score) before the observation period.
3. Outcomes:
4. Primary outcomes include survival rates, changes in the 40-item ALS assessment questionnaire (ALSAQ-40 score) and ALSFRS-R score, and any adverse events or serious adverse events.
5. Secondary outcomes: changes in forced vital capacity (FVC), grip strength in Kg and modified Norris scale score, and other adverse events (dysphagia, constipation, gait disturbance, insomnia, musculoskeletal disorders, and upper respiratory tract inflammation).

### Data analysis

The process of analyzing data was carried out through the utilization of Review Manager (RevMan) software version 5.4. The measure of effect utilized in the study was either risk ratio (RR) or mean difference (MD), along with 95% confidence intervals (CI), based on the nature of the data (dichotomous or continuous). We reported the significance at *P* value < 0.05. We assessed the heterogeneity using *I*-square (*I*^2^) and chi-square tests. Homogeneous data (*P* ≥ 0.1 or *I*^2^ < 50%) were pooled in a fixed-effect model, while heterogeneous data (*P* < 0.1 or *I*^2^ > 50%) were analyzed in a random-effect model. We performed subgroup analyses depending on the number of cycles and months of survival.

## Results

### Literature search

**Fig. 1:**
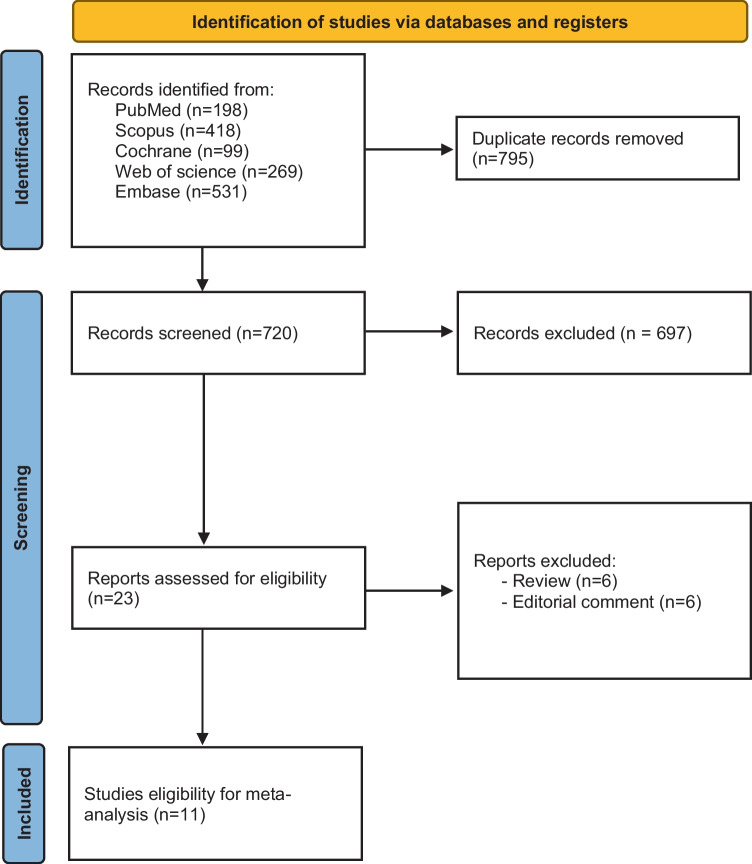
PRISMA flow chart

After removing duplicate results, the initial search returned a total of 720 unique articles. A subset of 23 studies were determined to be suitable for full-text assessment after undergoing a thorough screening process that involved evaluating the relevance of titles and abstracts. In the end, eleven articles fulfilled the inclusion criteria for our quantitative study after a thorough evaluation [18–21, 26, 28–33] (Fig. 1).

### Study characteristics

The studies included in this review comprised four RCTs [18–21] and seven retrospective cohort articles [26, 28–33]. Studies were conducted in the USA, Japan, Italy, Germany, and Iran. The total sample size was 2845 ALS patients, with a disease duration ranging from 1 to 2 years. There was quite a variety of ages represented among the people who took part in the research, with the youngest person being 55 years old and the oldest participant being 71 years old. In order to achieve comprehensive assessment of the outcomes being studied, we have included research works with varying follow-up durations, spanning from 1.5 to 40 months. Most patients were males and were administered riluzole (Tables 1 and 2**).**

**Table 1:** Summary of the included studies

ID	Study design	Trial registration	Follow-up	Site	Inclusion criteria	Main outcomes
Abe 2014	RCT	NCT00330681	24 W	Japan	- Patients with definite or probable ALS according to the House diagnostic criteria - Aged 20–75 yr - FVC > 70% - Duration of disease within 3 yr - A decrease of 1–4 points in the ALSFRS-R score - Graded 1 or 2 in the Japan ALS Severity Classification	ALSFRS-R, FVC, ALSAQ-40, and adverse events
Abe 2017	RCT	NCT01492686	24 W	Japan	- Patients aged 20 − 75 yr with definite or probable ALS according to the revised El Escorial criteria - Graded 1 or 2 in the Japan ALS Severity Classification - Scored at least 2 points of ALSFRS-R - FVC 80% or more - Disease duration of 2 yr or less - Had a decrease of 1 − 4 points in the ALSFRS-R score during a 12-week observation period	ALSFRS-R, FVC, ALSAQ-40, and adverse events
Abe 2017 (Grade 3)	RCT	NCT00415519	24 W	Japan	- Diagnosis of definite, probable, or probable laboratory-supported ALS - Grade 3 according to the Japan ALS severity classification - Age 20–75 yr - FVC of at least 60% - Duration of disease from the first symptom (any ALS symptom) within 3 yr - change in revised ALS functional rating scale (ALSFRS-R) (11,12) score during the 12-week pre-observation	ALSFRS-R, FVC, death, and adverse events
Brooks 2022	Retrospective cohort	NR	30 M	USA	- Patients with ALS, aged ≥ 18 yr - Diagnosis of ALS on any claim and in any inpatient or outpatient setting FVC at least 60% - Treated with IV edaravone vs controls with no edaravone administration - Patients may or may not have received treatment with riluzole	All-cause mortality
Fortuna 2019	Retrospective cohort	NR	6 M	North-Eastern Italy	- Patients with definite or probable ALS, aged 18–75 yr - A score of at least ≥ 2 per item of ALSFRS-R - FVC ≥ 80% of predicted - Disease duration ≤ 2 yr - A decrease of 1–4 points in the ALSFRS-R score	ALSFRS-R, FVC, and MRC
Houzen 2021	Retrospective cohort	NR	40 M	Japan	- Patients with definite or probable ALS - Treated at Obihiro Kosei Hospital between 2013 and 2018 - Treated with edaravone for an average duration of 26.6 (range, 2–64) months	Survival rate
Lunetta 2020	Retrospective cohort	NR	12 M	Italy	- Patients with definite or probable ALS - Patients who can eat a meal, excrete, or move alone - Patients of less than 2 yr after the onset of ALS - Patients whose progress of the condition during 12 weeks before administration meet other requirements FVC > 80%	ALSFRS-R and FVC
Okada 2018	Retrospective cohort	NR	12 M	Japan	- Patients with ALS were examined between 2010 and 2016 - All the patients before June 2015 were allocated to the control group - Patients were diagnosed with ALS according to the revised El Escorial criteria - FVC of at least 80% - Disease duration of ≤ 2 yr	ALSFRS-R, serum creatinine level, and survival rates
Eishi-Oskouei 2020	RCT	NCT03272802	12 M	Iran	- Patients with definite or probable ALS and graded as mild or moderate according to ALS Health State Scale - FVC of at least 80% - Age between 18 and 75 yr - Disease duration of ≤ 2 yr	ALSFRS-R and FVC
Vu 2020	Retrospective cohort	NR	6 M	USA	- Patients with ALS - One or more prescription fills of edaravone or riluzole - Data from August 1, 2017, to September 30, 2019 - FVC at least 80% - Disease duration of ≤ 2 yr	Death, hospitalization (ALS, dyspnea, or orthopnea), tracheostomy, mechanical ventilation, and survival rate
Witzel 2022	Retrospective cohort	NR	13 M	Germany	- Patients with probable or definite ALS according to the El Escorial criteria, aged 20–75 yr - Patients with definite or probable ALS, aged 18–75 yr - Scored ≥ 2 points of ALSFRS-R - Forced Vital Capacity (FVC) ≥ 80% of predicted - Disease duration ≤ 2 yr - A decrease of 1–4 points in the ALSFRS-R score through the 12-week observation period	ALSFRS-R

Abbreviations: *FVC* forced vital capacity, *ALSFRS-R* Amyotrophic Lateral Sclerosis Functional Rating Scale—revised, *ALSAQ-40* amyotrophic lateral sclerosis assessment questionnaire, *MRC* medical research council score

**Table 2:** Baseline characteristics of the included studies

ID	Arms	Number	Age, y	Male	ALS diagnosis definite	ALS diagnosis probable	ALS severity grade 1	Duration of disease, y	Riluzole use	ALSFRS-R*
Abe 2014	Edaravone	101	58 ± 7.3	63 (62.4%)	29 (28.7%)	52 (51.5%)	36 (35.6%)	1.3 ± 0.42	90 (89.1%)	43 ± 2.8
	Control	104	58.5 ± 7.8	69 (66.3%)	21 (20.2%)	54 (51.9%)	40 (38.5%)	1.2 ± 0.45	92 (88.5%)	44 ± 2.16
Abe 2017	Edaravone	69	60.5 ± 10	38 (55%)	28 (41%)	41 (59%)	22 (32%)	1.13 ± 0.5	63 (91%)	43.6 ± 2.2
	Control	68	60.1 ± 10	41 (60%)	27 (40%)	41 (60%)	16 (24%)	1.06 ± 0.5	62 (91%)	43.5 ± 2.2
Abe 2017 Grade 3	Edaravone	13	57.75 ± 6.65	7 (53.8%)	7 (53.8%)	4 (30.8%)	NR	1.625 ± 0.5	10 (76.9%)	34.75 ± 5
	Control	12	58.75 ± 7.82	6 (50%)	2 (16.7%)	8 (66.7%)	NR	2.025 ± 0.6	11 (91.7%)	36.5 ± 4
Brooks 2022	Edaravone	318	62.9 ± 10.1	184 (57.9%)	NR	NR	NR	NR	208 (65.4%)	NR
	Control	318	62.7 ± 10.2	184 (57.9%)	NR	NR	NR	NR	208 (65.4%)	NR
Fortuna 2019	Edaravone	31	65.0 ± 11.7	16 (52%)	NR	NR	NR	1.48 ± 0.5	31 (100%)	40.94 ± 3.63
	Control	50	60.5 ± 10.3	31 (62%)	NR	NR	NR	0.975 ± 0.48	46 (92%)	42.47 ± 2.34
Houzen 2021	Edaravone	22	64.8 ± 11.0	14 (64%)	NR	NR	NR	NR	18 (81.8%)	NR
	Control	23	71.4 ± 12.5	14 (61%)	NR	NR	NR	NR	14 (60.9%)	NR
Lunetta 2020	Edaravone	197	60 ± 2.3	111 (56.3%)	NR	NR	NR	NR	NR	NR
	Control	290	55 ± 2.58	187 (64.5%)	NR	NR	NR	NR	NR	NR
Okada 2018	Edaravone	27	62.0 ± 9.4	15 (56%)	NR	NR	2 (7%)	1.88 ± 1.275	25 (93%)	30.0 ± 12.1
	Control	30	67.2 ± 9.7	19 (63%)	NR	NR	2 (7%)	1.25 ± 0.96	23 (77%)	38.7 ± 6.3
Eishi-Oskouei 2020	Edaravone	10	55.20 ± 13.50	9 (90%)	NR	NR	NR	NR	NR	NR
	Control	10	59.20 ± 12.92	8 (80%)	NR	NR	NR	NR	NR	NR
Vu 2020	Edaravone	223	64.6 ± 11.3	216 (96.9%)	NR	NR	NR	NR	157 (70.4%)	39 ± 3.7
	Control	669	NR	649 (97%)	NR	NR	NR	NR	669 (100%)	34.67 ± 6.68
Witzel 2022	Edaravone	130	57.5 ± 10.5	82 (63%)	NR	NR	NR	1.375 ± 0.8	NR	37.6 ± 7
	Control	130	56.7 ± 10.5	83 (64%)	NR	NR	NR	1.38 ± 0.88	123 (96%)	38.6 ± 5.2

Abbreviations: *NR* not reported, *ALS* amyotrophic lateral sclerosis, *ALSFRS-R* Amyotrophic Lateral Sclerosis Rating Scale, revised

The score was measured before the observation period

Data were presented as mean ± standard deviation or numbers (percentages)

### The quality of the included studies

**Fig. 2:**
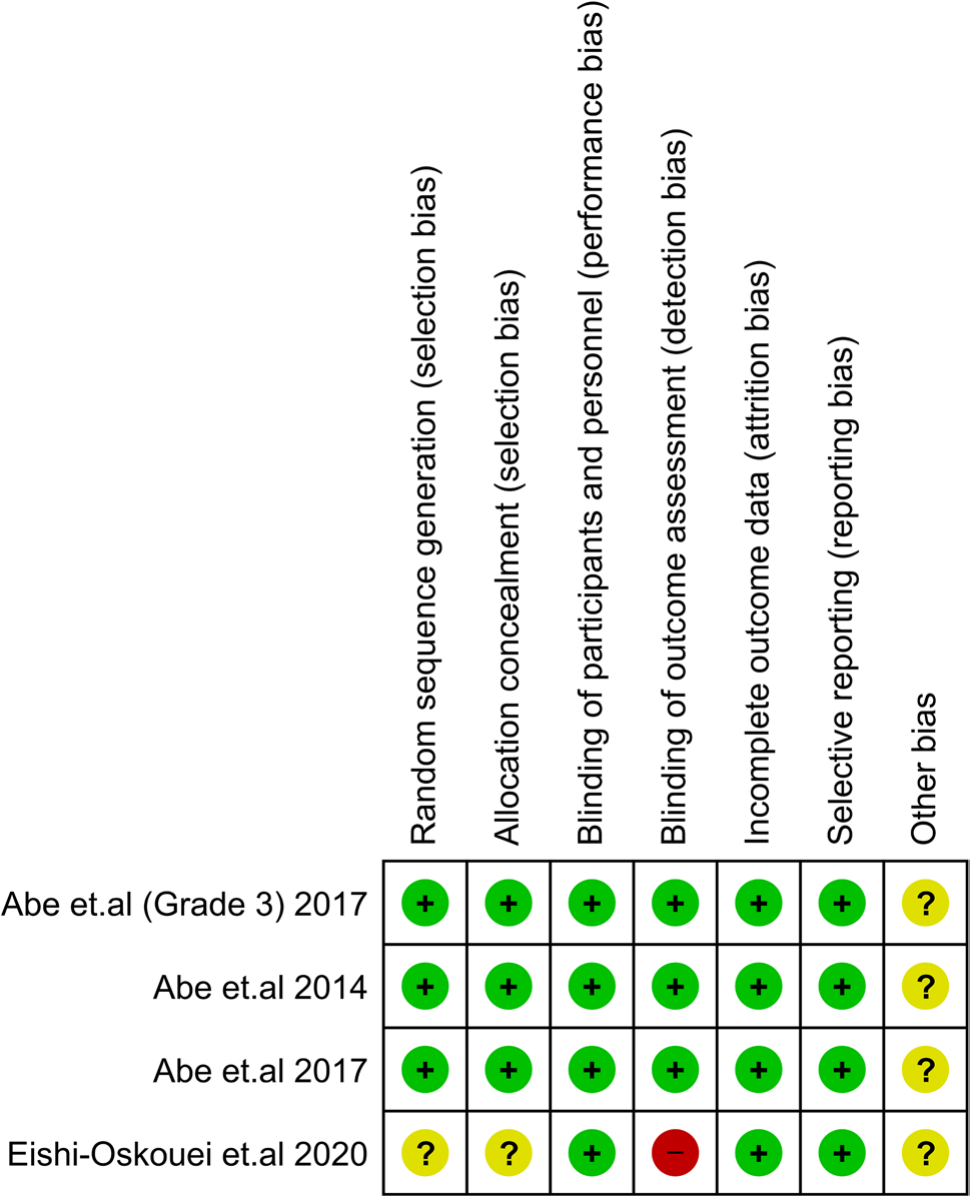
Risk of bias summary for RCTs

The quality of the RCTs that were incorporated in our analysis was rigorously evaluated to ensure the dependability and accuracy of our results. The majority of the RCTs exhibited a low probability of bias, suggesting a considerable degree of methodological quality and a reliable basis for our analysis. Upon further examination, it was discovered that the study conducted by Eishi-Oskouei et al. exhibited a higher probability of bias [21], which revealed an unclear selection bias risk and a high detection bias risk **(**Fig. 2**)**. Our cohort studies had a fair quality; the details are in **Online Resource **1. Using the GRADE method, the quality of the evidence in its entirety was ranked as being somewhere between moderate and very low **(Online Resource **2**)**.

### Primary outcomes

#### Survival rate

**Fig. 3:**
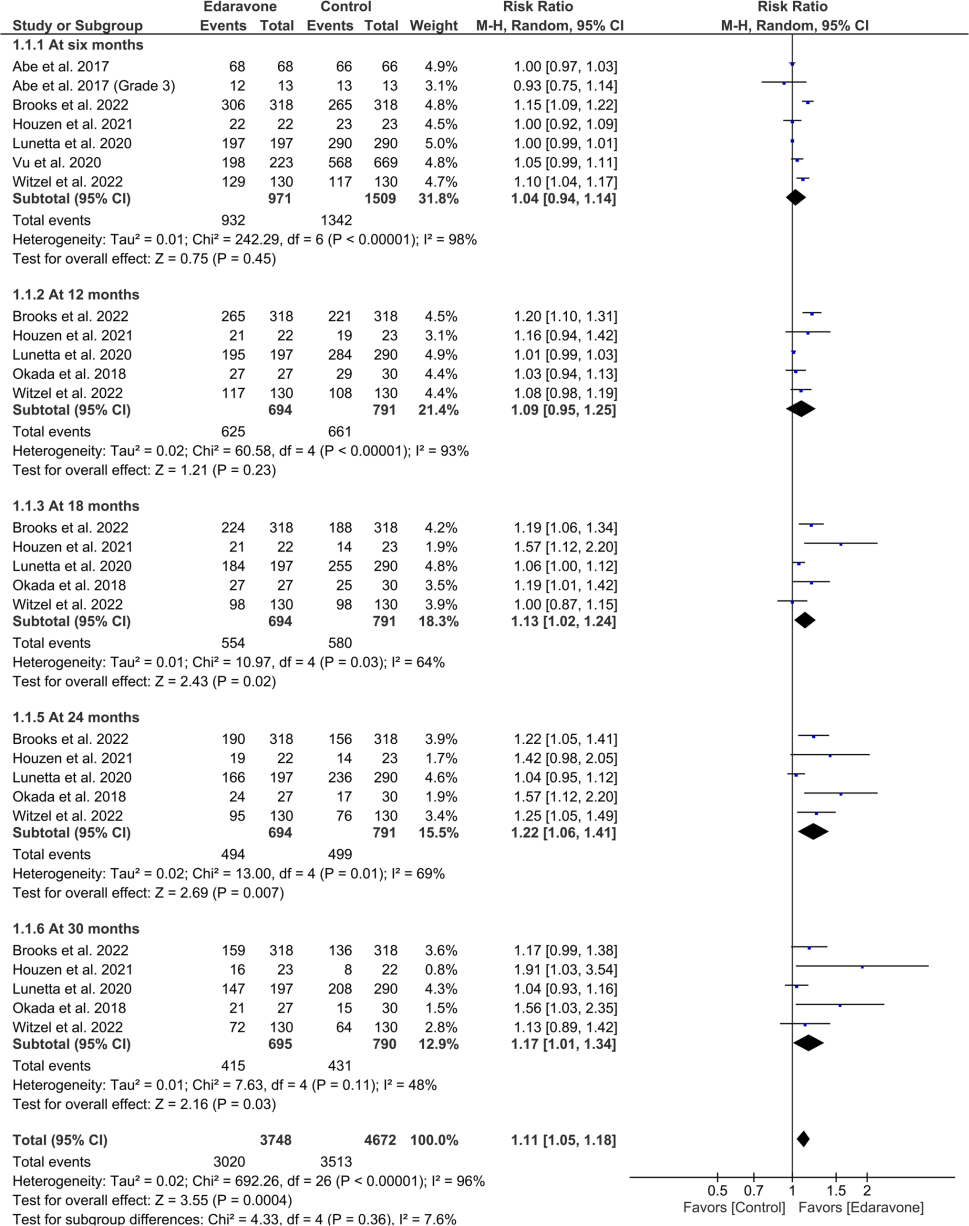
A forest plot of the survival rates

Our findings indicate that edaravone exhibited a favorable outcome in comparison to the control group (RR = 1.11, 95% CI [1.05 to 1.18], *P* = 0.0004). The results obtained through pooling of data were found to be heterogeneous (*P* < 0.001, *I*^2^ = 96%) (Fig. 3).

##### At 6 months

Seven studies with 2480 ALS patients reported survival rates at 6 months. The results were insignificant (RR = 1.04, 95% CI [0.94 to 1.14], *P* value = 0.45) and heterogeneous (*P* < 0.001, *I*^2^ = 98%) (Fig. 3).

##### At 12 months

The pooled data of six articles with 1485 ALS patients revealed an insignificant difference between the two groups (RR = 1.09, 95% CI [0.95 to 1.25], *P* = 0.23). The results obtained through pooling of data were found to be heterogeneous (*P* < 0.001, *I*^2^ = 93%) **(**Fig. 3).

##### At 18 months

Our findings indicate that the edaravone group exhibited a statistically significant increase in survival rate compared to the control group, as observed in a meta-analysis of five studies involving a total of 1485 patients diagnosed with ALS (RR = 1.13, 95% CI [1.02 to 1.24], *P* = 0.02). The results obtained through pooling of data were found to be heterogeneous (*P* = 0.03, *I*^2^ = 64%) (Fig. 3).

##### At 24 months

The survival rate at 24 months was reported in five studies with 1485 ALS patients. The results significantly favored the edaravone group over the control group (RR = 1.22, 95% CI [1.06 to 1.41], *P* = 0.007). The results obtained through pooling of data were found to be heterogeneous (*P* = 0.01, *I*^2^ = 69%) (Fig. 3).

##### At 30 months

Five studies were pooled with a sample size of 1485. The survival rate at 30 months was higher in the edaravone group (RR = 1.17, 95% CI [1.01 to 1.34], *P* = 0.03). The results obtained through pooling of data were found to be homogeneous (*P* = 0.11, *I*^2^ = 48%) (Fig. 3).

#### Change in ALSAQ 40 score

**Fig. 4:**
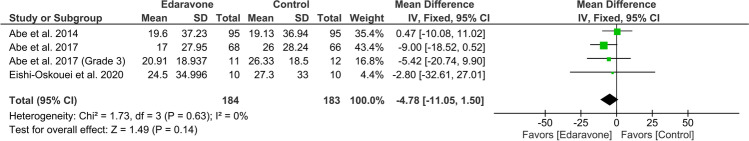
A forest plot of the change in ALSAQ 40 score

The pooled estimate (sample size = 367) showed no difference between both groups (MD =  − 4.78, 95% CI [− 11.05 to 1.5], *P* = 0.14). The results obtained through pooling of data were found to be homogeneous (*P* = 0.63, *I*^2^ = 0%) (Fig. 4).

#### Change in ALSFRS-R score

**Fig. 5:**
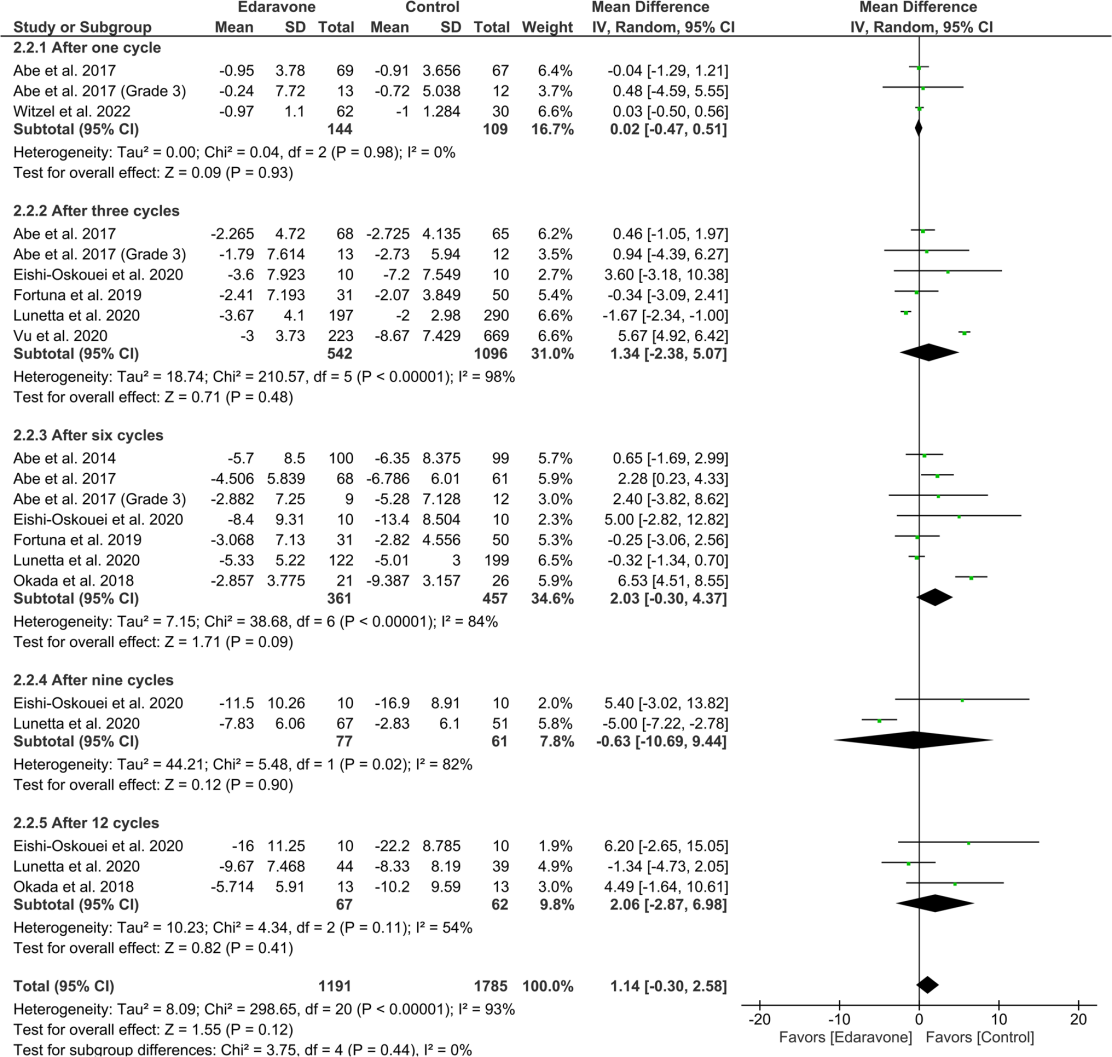
A forest plot of the change in ALSFRS-R score

The overall effect estimate showed no difference in ALSAQ 40 score between both groups (MD = 1.14, 95% CI [− 0.30 to 2.58], *P* = 0.12). The results obtained through pooling of data were found to be heterogeneous (*P* < 0.00001, *I*^2^ = 93%) (Fig. 5).

##### After one cycle

Three studies reported this score after the first cycle with a sample size of 253 ALS patients. There was insignificant variation between the groups (MD = 0.02, 95% CI [− 0.47 to 0.51], *P* = 0.93). The results were homogeneous (*P* = 0.98, *I*^2^ = 0%) (Fig. 5).

##### After three cycles

Six studies with 1638 ALS patients mentioned this outcome. There were insignificant results (MD = 1.34, 95% CI [− 2.38 to 5.07], *P* = 0.48). The results obtained through pooling of data were found to be heterogeneous (*P* < 0.001, *I*^2^ = 98%) (Fig. 5).

##### After six cycles

The pooled data of seven articles with 818 ALS patients showed insignificant results (MD = 2.03, 95% CI [− 0.30 to 4.37], *P* = 0.09). The results obtained through pooling of data were found to be heterogeneous (*P* < 0.001, *I*^2^ = 84%) (Fig. 5).

##### After nine cycles

The analysis of 138 ALS patients revealed insignificant results (MD =  − 0.63, 95% CI [− 10.69 to 9.44], *P* = 0.90). The studies were heterogeneous (*P* = 0.02, *I*^2^ = 82%) (Fig. 5).

##### After 12 cycles

The results of three studies with 129 ALS patients were insignificant (MD = 2.06, 95% CI [− 2.87 to 6.98], *P* = 0.41). The results obtained through pooling of data were found to be homogeneous (*P* = 0.11, *I*^2^ = 54%) (Fig. 5).

### Any adverse event

**Fig. 6:**
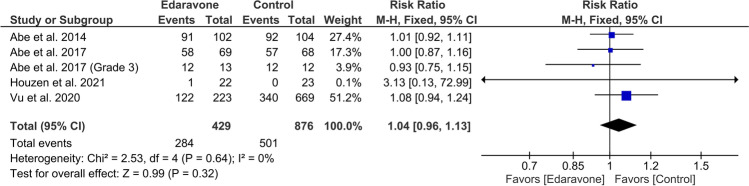
A forest plot of any adverse events

Five articles (sample size = 1305) revealed insignificant variation between both groups (RR = 1.04, 95% CI [0.96 to 1.13], *P* = 0.32). The results obtained through pooling of data were found to be homogeneous (*P* = 0.64, *I*^2^ = 0%) (Fig. 6).

### Serious adverse events

**Fig. 7:**
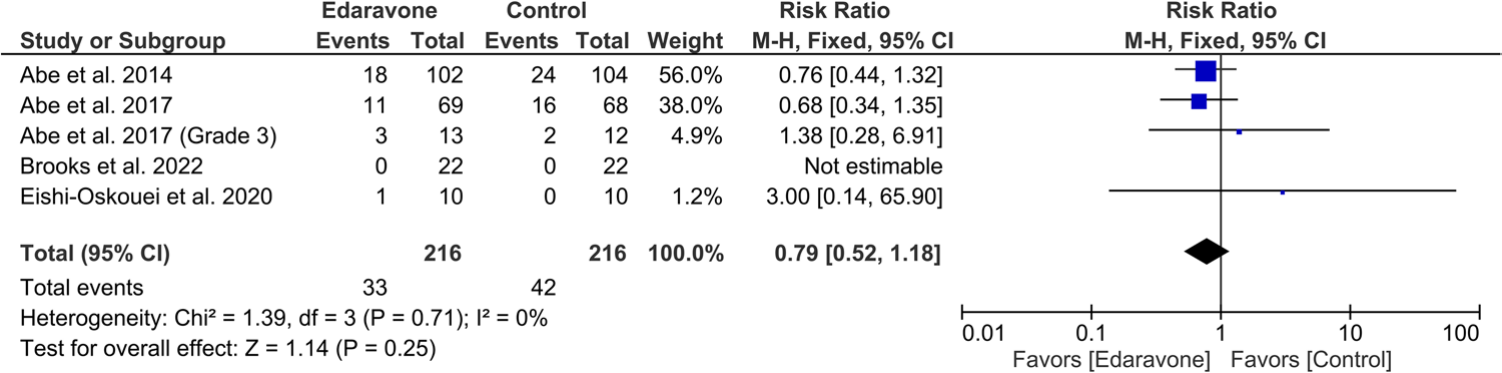
A forest plot of serious adverse events

There were five studies total that documented serious adverse effects. The pooled data showed insignificant results (RR = 0.79, 95% CI [0.52 to 1.18], *P* = 0.25). The results obtained through pooling of data were found to be homogeneous (*P* = 0.71, *I*^2^ = 0%) (Fig. 7).

### Secondary outcomes

#### Change in FVC (%)

Five articles reported the FVC for 782 ALS patients. There was no improvement in FVC score in ALS patients treated with edaravone (MD = 0.02, 95% CI [− 5.69 to 5.74], *P* = 0.99). Heterogeneity was observed (*P* = 0.008, *I*^2^ = 71%) **(Online Resource 3; **Fig. A1**)**.

#### Change in modified Norris scale

The analyzed data (sample size = 350) showed no variation between the edaravone and control groups (MD = 2.93, 95% CI [− 0.82 to 6.69], *P* = 0.13). The results obtained through pooling of data were found to be homogeneous (*P* = 0.63, *I*^*2*^ = 0%) **(Online Resource 3; **Fig. A2**)**.

#### Change in grip strength (kg)

Three studies reported this outcome with 358 ALS patients. There was an insignificant difference between both groups (MD = 0.44, 95% CI [− 0.69 to 1.57], *P* = 0.44). The analysis showed homogeneity (*P* = 0.81, *I*^2^ = 0%) (Online Resource 3; Fig. A3).

#### Change in pinch strength

The pooled data of 358 ALS patients revealed insignificant differences (MD = 0.09, 95% CI [− 0.17 to 0.35], *P* = 0.50). The data were homogeneous (*P* = 0.57, *I*^2^ = 0%) **(Online Resource 3; **Fig. A4**)**.

#### Other adverse events

The results showed that the edaravone-treated patients had significantly lower musculoskeletal disorders than the control group (RR = 0.84, 95% CI [0.74 to 0.95], *P* = 0.005). Our results were homogeneous (*P* = 0.96, *I*^2^ = 0%) **(Online Resource 3; **Fig. A5**)**. Other adverse events such as gait disturbance, dysphagia, constipation, insomnia, and upper respiratory tract inflammation showed insignificant results (*P* > 0.05). Moreover, the most commonly reported adverse events were gait disturbance [18, 20, 21, 28] and dysphagia [18, 19, 21, 28], reported by four studies with a total of 160 events out of 443 patients in the edaravone-treated patients and 183 out of 444 patients in the control group regarding gait disturbance and 154 out of 499 in the edaravone group and 160 out of 500 in the control group regarding the dysphagia. Nevertheless, we found adverse events with lower incidence than the above such as constipation reported by three studies [18, 19, 21] and insomnia reported by two studies [18, 19] with a total of 24 constipation events out of 181, and 14 insomnia events out of 171 in the edaravone group, and 25 out of 182, and 14 out of 172 in the control group respectively. Finally, upper respiratory tract inflammation was only reported by two studies [19, 20] with a total of 8 events out of 82 patients in the edaravone-treated group, and 3 events out of 80 patients in the control group A10**(Online Resource 3; **Fig. A6**-**A10**).**

### Sensitivity analysis

The survival rates at 18 and 24 months were homogeneous after excluding Houzen et al. (2021) [31] (*P* = 0.1) and Lunetta et al. (2020) [32] (*P* = 0.53), respectively. The results remained significant (*P* < 0.05) **(Online Resource 3; **Fig. A11**)**. The heterogeneity of the change in ALSFRS-R score after six cycles was solved by removing Okada et al. (2018) [33] (*P* = 0.22). The results remained insignificant (*P* = 0.3) **(Online Resource 3; **Fig. A12**)**. The results of FVC were homogeneous (*P* = 0.77) and still insignificant (*P* = 0.12) after sensitivity analysis **(Online Resource 3; **Fig. A13**).**

## Discussion

Our analysis showed that edaravone improved ALS patients’ survival rates after 18, 24, and 30 months compared to the control group. Moreover, edaravone was generally safe and associated with fewer musculoskeletal disorders than the control group. None of the other assessed outcomes differed significantly between the two groups. The aforementioned findings have the potential to guide forthcoming investigations and inform medical professionals’ clinical judgements concerning the administration of edaravone in relation to these particular outcomes.

A limited number of our included studies reported the dropout rate for some reasons. Witzel et al. reported that 6.9% of patients treated with edaravone died before the follow-up endpoint and 3.8% of patients discontinued therapy before follow-up in the same group [29]. Also, Abe et al. mentioned that eight out of 68 patients assigned to the placebo group discontinued due to different reasons; four for adverse events, two withdrew their consent, one had a tracheotomy, and one because of the investigator’s decision [19]. Furthermore, Abe et al. reported that only two patients out of 69 patients assigned to the edaravone group discontinued treatment; one was due to respiratory abnormalities and the other had a tracheotomy [19]. Abe et al. (Grade 3) 2017 reported that four patients discontinued treatment in the edaravone-treatment group; two patients due to their desire, one because of adverse events, and the last one because of worsening ALS that made the hospital visit very hard [20]. However, most of the included studies have addressed this issue by using the intention to treat analysis method accounting for any dropout.

Patients with ALS had a 3-year median survival time after first experiencing symptoms [34]. Current survival data of edaravone were contradictory and were mainly reported in retrospective studies with most studies showing the beneficial effect of Edaravone in overall survival rates [28, 31, 33], which is similar to our analysis results that showed improved survival rates measured at 18, 24, and 30 months. However, in contrast to our results Lunetta et al. and Witel et al. reported no significant difference with Edaravone in survival rates measured up to D-50 and 33 months respectively. [29, 32]

The major purpose of this research was to determine if edaravone is effective for treating ALS, with a particular emphasis on the ALSFRS-R score. ALSFRS-R is a revised version of the ALSFRS, and it encompasses an assessment of respiratory function and has been found to exhibit a significant correlation with the quality of life of a patient [35]. Among those diagnosed with early-stage ALS, edaravone has been shown to be effective in eliciting significant improvements in the ALSFRS-R score compared to a placebo [19]. In contrast to a prior meta-analysis [17], we found no evidence of a significant improvement in ALSFRS-R scores after giving edaravone to a larger sample of ALS patients. The aforementioned results could hold significant implications for the use of edaravone as a therapeutic option for ALS and highlight the necessity for additional investigation in this domain to gain an in-depth understanding of the possible advantages of this intervention.

The results of our study suggest that there was insufficient statistical significance observed in the incidence of most adverse events when comparing both groups, which was also similar to the findings of the last meta-analysis [17]; however, we found significantly fewer musculoskeletal disorders with Edaravone.

Our study provides an updated analysis of the recently published data on the safety and efficacy of Edaravone in ALS. Moreover, this is the first meta-analysis to address survival outcomes. On the other hand, our study had several limitations. First, it included patients who were already taking riluzole and did not consider any interactions between both drugs. Second, many studies did not report the grade of the disease [21, 26, 28–33]; therefore, we cannot know the drug’s effect on different stages of the disease. Third, we have data from retrospective studies, which may be affected by selection bias. And finally, the pooled analysis data showed significant heterogeneity in most of the assessed outcomes.

## Conclusion

According to our results, the administration of edaravone could potentially improve the survival rates of individuals diagnosed with amyotrophic lateral sclerosis at the 18, 24, and 30-month intervals relative to the control group. Additionally, the use of edaravone appears to be linked with a lower incidence of musculoskeletal disorders, but other assessed outcomes, such as the ALSFRS-R scores, exhibited no significant differences between the two groups. However, more research is required to ascertain its effectiveness in particular patient cohorts and stages of illness.

## Supplementary Information

Below is the link to the electronic supplementary material.

Supplementary file1 (DOCX 20 KB)

Supplementary file2 (DOCX 19 KB)

Supplementary file3 (PDF 256 KB)

## Data Availability

The data supporting this study’s findings are available on request from the corresponding author.
