# Supplementary material for: Safety and efficacy of edaravone in patients with amyotrophic lateral sclerosis: a systematic review and meta-analysis
Source: Neurol Sci. 2023 May 30;44(10):3429–42. doi: 10.1007/s10072-023-06869-8 (PMC10495275; doi:10.1007/s10072-023-06869-8)
Supplement: Supplementary file 1 — Supplementary file1 (DOCX 20 KB) [file 10072_2023_6869_MOESM1_ESM.docx]

**Title: Safety and Efficacy of Edaravone in Patients with Amyotrophic Lateral Sclerosis: A Systematic Review and Meta-analysis.**

**Authors:** Anas Zakarya Nourelden^#1^, Ibrahim Kamal^#1^, Abdulrahman Ibrahim Hagrass^1*^, Abdelrahman G. Tawfik^2^, Mahmoud M. Elhady^3^, Ahmed Hashem Fathallah^*^, Mona Muhe Eldeen Eshag^5^ Mohamed Sayed Zaazouee^6^

**Affiliations**

^1^Faculty of Medicine, Al-Azhar University, Cairo, Egypt

^2^Department of Pharmacotherapy, College of Pharmacy, The University of Utah, Salt Lake City, UT, USA

^3^Faculty of Medicine, Benha University, Qalubiya, Egypt

^4^Faculty of Medicine, Minia University, Minia, Egypt

^5^Faculty of Medicine, University of Bahri, Khartoum, Sudan

^6^Faculty of Medicine, Al-Azhar University, Assiut, Egypt

^#^Both authors equally contributed to the study.

**Journal name:** *Neurological Sciences*
***Correspondence:** Abdulrahman Ibrahim Hagrass; Abdulrahmanelsayed.stu.3@azhar.edu.eg; Tel.: +201010344694; Address: New Cairo, Cairo Governorate, Egypt; ORCID: <https://orcid.org/0000-0002-0297-9385>.

**Supplemental Table 1:** Quality assessment of the observational studies.

| ID | NIH Quality Assessment Tool for Observational Cohort and Cross-Sectional Studies | | | | | | | | | | | | | | | |
| --- | --- | --- | --- | --- | --- | --- | --- | --- | --- | --- | --- | --- | --- | --- | --- | --- |
|  | 1 | 2 | 3 | 4 | 5 | 6 | 7 | 8 | 9 | 10 | 11 | 12 | 13 | 14 | Total scores | Quality rating |
| Brooks et.al 2022 | Yes | YES | Yes | Yes | Yes | NA | Yes | NR | Yes | NR | Yes | NR | Yes | Yes | 10 | Fair |
| Witzel et.al 2021 | Yes | Yes | Yes | Yes | NR | NA | Yes | NR | Yes | NR | Yes | NR | No | Yes | 8.5 | Fair |
| Fortuna et.al 2019 | Yes | Yes | Yes | Yes | NR | NA | Yes | NR | Yes | NR | NR | NR | Yes | Yes | 8 | Fair |
| Houzen et.al 2021 | Yes | Yes | Yes | Yes | No | NA | Yes | NR | Yes | NR | NR | NR | Yes | Yes | 8.5 | Fair |
| Lunetta et.al 2020 | Yes | Yes | Yes | Yes | NR | NA | Yes | NR | Yes | NR | NR | NR | Yes | Yes | 8 | Fair |
| Okada et.al 2018 | Yes | Yes | CD | Yes | No | NA | Yes | NR | Yes | NR | NR | NR | Yes | Yes | 7.5 | Fair |
| Vu et.al 2020 | Yes | Yes | Yes | Yes | Yes | NA | Yes | NR | Yes | NR | NR | NR | Yes | Yes | 9 | Fair |

1. Was the research question or objective in this paper clearly stated?

2. Were eligibility/selection criteria for the study population prespecified and clearly described?

3. Were the participants in the study representative of those who would be eligible for the test/service/intervention in the general or clinical population of interest?

4. Were all eligible participants that met the prespecified entry criteria enrolled?

5. Was the sample size sufficiently large to provide confidence in the findings?

6. For the analyses in this paper, were the exposure(s) of interest measured prior to the outcome(s) being measured?

7. Was the time frame sufficient so that one could reasonably expect to see an association between exposure and outcome if it existed?

8. For exposures that can vary in amount or level, did the study examine different levels of the exposure as related to the outcome (eg, categories of exposure, or exposure measured as continuous variable)?

9. Were the exposure measures (independent variables) clearly defined, valid, reliable, and implemented consistently across all study participants?

10. Was the exposure(s) assessed more than once over time?

11. Were the outcome measures prespecified, clearly defined, valid, reliable, and assessed consistently across all study participants?

12. Were the people assessing the outcomes blinded to the participants' exposures/interventions?

13. Was the loss to follow-up after baseline 20% or less? Were those lost to follow-up accounted for in the analysis?

14.Were key potential confounding variables measured and adjusted statistically for their impact on the relationship between exposure(s) and outcome(s)?

Scores: Yes = 1 // No = 0.5 // NR & NA & CD = 0

Quality rating: good (11-14 points) or fair (7.5-10.5 points) or poor (0-7 points).
