# Supplementary material for: Safety and efficacy of edaravone in patients with amyotrophic lateral sclerosis: a systematic review and meta-analysis
Source: Neurol Sci. 2023 May 30;44(10):3429–42. doi: 10.1007/s10072-023-06869-8 (PMC10495275; doi:10.1007/s10072-023-06869-8)
Supplement: Supplementary file 2 — Supplementary file2 (DOCX 19 KB) [file 10072_2023_6869_MOESM2_ESM.docx]

**Title: Safety and Efficacy of Edaravone in Patients with Amyotrophic Lateral Sclerosis: A Systematic Review and Meta-analysis.**

**Authors:** Anas Zakarya Nourelden^#1^, Ibrahim Kamal^#1^, Abdulrahman Ibrahim Hagrass^1*^, Abdelrahman G. Tawfik^2^, Mahmoud M. Elhady^3^, Ahmed Hashem Fathallah^*^, Mona Muhe Eldeen Eshag^5^ Mohamed Sayed Zaazouee^6^

**Affiliations**

^1^Faculty of Medicine, Al-Azhar University, Cairo, Egypt

^2^Department of Pharmacotherapy, College of Pharmacy, The University of Utah, Salt Lake City, UT, USA

^3^Faculty of Medicine, Benha University, Qalubiya, Egypt

^4^Faculty of Medicine, Minia University, Minia, Egypt

^5^Faculty of Medicine, University of Bahri, Khartoum, Sudan

^6^Faculty of Medicine, Al-Azhar University, Assiut, Egypt

^#^Both authors equally contributed to the study.

**Journal name:** *Neurological Sciences*
***Correspondence:** Abdulrahman Ibrahim Hagrass; Abdulrahmanelsayed.stu.3@azhar.edu.eg; Tel.: +201010344694; Address: New Cairo, Cairo Governorate, Egypt; ORCID: <https://orcid.org/0000-0002-0297-9385>.

**Supplemental Table 2:** Quality assessment of the observational studies.

| **Certainty assessment** | | | | | | **№ of patients** | | **Effect** | | | **Certainty** | | **Importance** |
| --- | --- | --- | --- | --- | --- | --- | --- | --- | --- | --- | --- | --- | --- |
| **№ of studies** | **Risk of bias** | **Inconsistency** | **Indirectness** | **Imprecision** | **Other considerations** | **Edaravone** |  | **Relative (95% CI)** | | **Absolute (95% CI)** |  |  | |
| **Change at ALSFRS-R score** | | | | | | | | | | | | | |
| 9 | serious | very serious | not serious | serious | none | 1191 | 1785 | - | | MD **1.14 higher** (0.3 lower to 2.58 higher) | ⨁◯◯◯ Very low | CRITICAL | |
| **Change at ALSAQ-40 score** | | | | | | | | | | | | | |
| 4 | not serious | not serious | not serious | serious | none | 184 | 183 | - | | MD **4.78 lower** (11.05 lower to 1.5 higher) | ⨁⨁⨁◯ Moderate | IMPORTANT | |
| **Change at FVC(%)** | | | | | | | | | | | | | |
| 4 | not serious | very serious | not serious | serious | none | 240 | 343 | - | | MD **8.87 lower** (17.02 lower to 0.73 lower) | ⨁◯◯◯ Very low | IMPORTANT | |
| **Survival rate** | | | | | | | | | | | | | |
| 8 | serious | very serious | not serious | not serious | none | 3080/3817 (80.7%) | 3567/4740 (75.3%) | **RR 1.11** (1.05 to 1.18) | | **83 more per 1,000** (from 38 more to 135 more) | ⨁◯◯◯ Very low | CRITICAL | |
| **Survival rate - 6 months** | | | | | | | | | | | | | |
| 7 | serious | very serious | not serious | serious | none | 932/971 (96.0%) | 1342/1509 (88.9%) | | **RR 1.04** (0.94 to 1.14) | **36 more per 1,000** (from 53 fewer to 125 more) | ⨁◯◯◯ Very low | CRITICAL | |
| **Survival rate - 12 months** | | | | | | | | | | | | | |
| 6 | serious | very serious | not serious | serious | none | 685/763 (89.8%) | 715/859 (83.2%) | | **RR 1.09** (0.97 to 1.23) | **75 more per 1,000** (from 25 fewer to 191 more) | ⨁◯◯◯ Very low | CRITICAL | |
| **Survival rate - 18 months** | | | | | | | | | | | | | |
| 5 | serious | serious | not serious | not serious | none | 554/694 (79.8%) | 580/791 (73.3%) | | **RR 1.13** (1.02 to 1.24) | **95 more per 1,000** (from 15 more to 176 more) | ⨁◯◯◯ Very low | CRITICAL | |
| **Survival rate - 24 months** | | | | | | | | | | | | | |
| 5 | serious | serious | not serious | not serious | none | 494/694 (71.2%) | 499/791 (63.1%) | | **RR 1.22** (1.06 to 1.41) | **139 more per 1,000** (from 38 more to 259 more) | ⨁◯◯◯ Very low | CRITICAL | |
| **Survival rate - 30 months** | | | | | | | | | | | | | |
| 5 | serious | serious | not serious | not serious | none | 415/695 (59.7%) | 431/790 (54.6%) | | **RR 1.17** (1.01 to 1.34) | **93 more per 1,000** (from 5 more to 185 more) | ⨁◯◯◯ Very low | CRITICAL | |
| **Adverse events** | | | | | | | | | | | | | |
| 5 | not serious | not serious | not serious | serious | none | 284/429 (66.2%) | 501/876 (57.2%) | | **RR 1.04** (0.96 to 1.13) | **23 more per 1,000** (from 23 fewer to 74 more) | ⨁⨁⨁◯ Moderate | IMPORTANT | |
| **Serious adverse events** | | | | | | | | | | | | | |
| 5 | not serious | not serious | not serious | serious | none | 33/216 (15.3%) | 42/216 (19.4%) | | **RR 0.79** (0.52 to 1.18) | **41 fewer per 1,000** (from 93 fewer to 35 more) | ⨁⨁⨁◯ Moderate | IMPORTANT | |

**CI:** confidence interval; **MD:** mean difference; **RR:** risk ratio.
