# Supplementary material for: Safety and efficacy of edaravone in patients with amyotrophic lateral sclerosis: a systematic review and meta-analysis
Source: Neurol Sci. 2023 May 30;44(10):3429–42. doi: 10.1007/s10072-023-06869-8 (PMC10495275; doi:10.1007/s10072-023-06869-8)
Supplement: Supplementary file 3 — Supplementary file3 (PDF 256 KB) [file 10072_2023_6869_MOESM3_ESM.pdf]

Title: Safety and Efficacy of Edaravone in Patients with Amyotrophic Lateral Sclerosis: A Systematic Review and Meta-analysis.

Authors: Anas Zakarya Nourelden[1#], Ibrahim Kamal[1#], Abdulrahman Ibrahim Hagrass[1*], Abdelrahman G. Tawfik[2], Mahmoud M. Elhady[3], Ahmed Hashem Fathallah[4], Mona Muhe Eldeen Eshag[5] Mohamed Sayed Zaazouee[6]

Affiliations

[1]Faculty of Medicine, Al-Azhar University, Cairo, Egypt

[2]Department of Pharmacotherapy, College of Pharmacy, The University of Utah, Salt Lake City, UT, USA

[3]Faculty of Medicine, Benha University, Qalubiya, Egypt

[4]Faculty of Medicine, Minia University, Minia, Egypt

[5]Faculty of Medicine, University of Bahri, Khartoum, Sudan

[6]Faculty of Medicine, Al-Azhar University, Assiut, Egypt

[#]Both authors equally contributed to the study.

Journal name: *Neurological Sciences*

***Correspondence:** Abdulrahman Ibrahim Hagrass; Abdulrahmanelsayed.stu.3@azhar.edu.eg; Tel.: +201010344694; Address: New Cairo, Cairo Governorate, Egypt; ORCID: https://orcid.org/0000-0002-0297-9385.

Study or Subgroup	Edaravone Mean	Edaravone SD	Edaravone Total	Control Mean	Control SD	Control Total	Weight	Mean Difference IV, Random, 95% CI
Abe et al. 2014	-14.57	24.1	100	-17.49	23.78	99	21.2%	2.92 [-3.73, 9.57]
Abe et al. 2017	-15.61	19.87	68	-20.4	20.147	66	21.0%	4.79 [-1.99, 11.57]
Abe et al. 2017 (Grade 3)	-18.75	16.51	13	-15.69	15.865	12	12.1%	-3.06 [-15.75, 9.63]
Fortuna et al. 2019	-13.9	15.9	31	-17	18	50	19.7%	3.10 [-4.40, 10.60]
Lunetta et al. 2020	-16.167	20.99	128	-9.067	12.746	215	26.0%	-7.10 [-11.12, -3.08]
**Total (95% CI)**			**340**			**442**	**100.0%**	**0.02 [-5.69, 5.74]**

Heterogeneity: Tau² = 28.55; Chi² = 13.94, df = 4 (P = 0.008); I² = 71%
Test for overall effect: Z = 0.01 (P = 0.99)

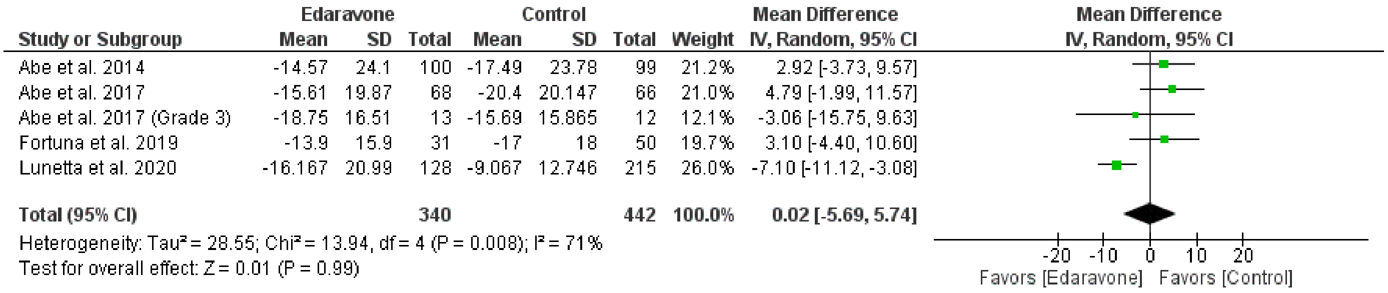

Fig. A1 which demonstrates the pooled mean difference of the Change in FVC.

Study or Subgroup	Edaravone Mean	Edaravone SD	Edaravone Total	Control Mean	Control SD	Control Total	Weight	Mean Difference IV, Fixed, 95% CI
Abe et al. 2014	-14.12	19.98	95	-16.15	19.697	97	44.8%	2.03 [-3.58, 7.64]
Abe et al. 2017	-15.91	16.245	68	-20.8	17.735	66	42.5%	4.89 [-0.87, 10.65]
Abe et al. 2017 (Grade 3)	-18.18	13.16	12	-17.76	13.16	12	12.7%	-0.42 [-10.95, 10.11]
**Total (95% CI)**			**175**			**175**	**100.0%**	**2.93 [-0.82, 6.69]**

Heterogeneity: Chi² = 0.93, df = 2 (P = 0.63); I² = 0%
Test for overall effect: Z = 1.53 (P = 0.13)

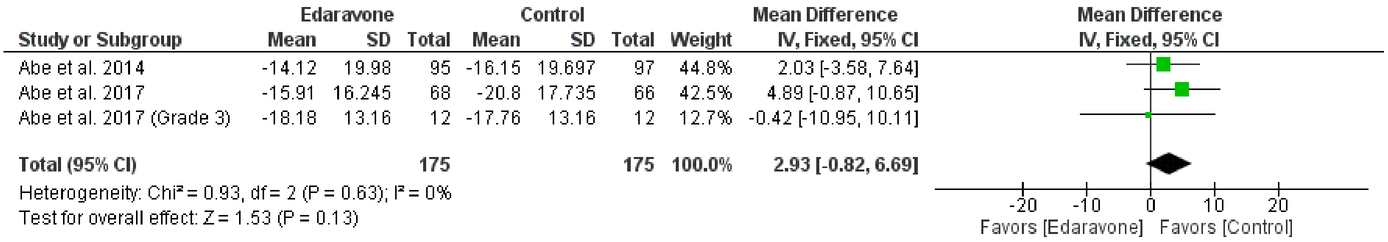

Fig. A2 which demonstrates the pooled mean difference of the Change in modified Norris scale.

Study or Subgroup	Edaravone Mean	Edaravone SD	Edaravone Total	Control Mean	Control SD	Control Total	Weight	Mean Difference IV, Fixed, 95% CI
Abe et al. 2014	-4.81	6.9	100	-5.71	6.865	99	34.9%	0.90 [-1.01, 2.81]
Abe et al. 2017	-4.08	4.45	68	-4.19	4.55	66	54.9%	0.11 [-1.41, 1.63]
Abe et al. 2017 (Grade 3)	-3.06	4.465	13	-3.72	4.538	12	10.2%	0.66 [-2.87, 4.19]
**Total (95% CI)**			**181**			**177**	**100.0%**	**0.44 [-0.69, 1.57]**

Heterogeneity: Chi² = 0.42, df = 2 (P = 0.81); I² = 0%
Test for overall effect: Z = 0.77 (P = 0.44)

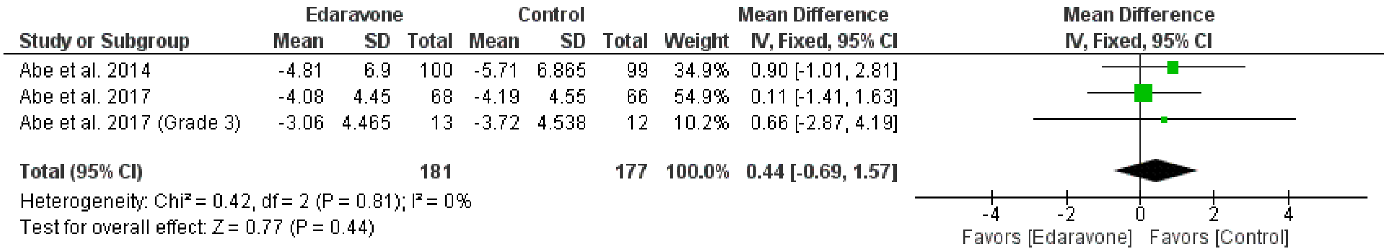

Fig. A3 which demonstrates the pooled mean difference of the Change in grip strength.

Study or Subgroup	Edaravone Mean	Edaravone SD	Edaravone Total	Control Mean	Control SD	Control Total	Weight	Mean Difference IV, Fixed, 95% CI
Abe et al. 2014	-0.83	1.5	100	-1.03	1.49	99	39.7%	0.20 [-0.22, 0.62]
Abe et al. 2017	-0.78	1.154	68	-0.88	1.137	66	45.5%	0.10 [-0.29, 0.49]
Abe et al. 2017 (Grade 3)	-0.5	0.865	13	-0.27	0.866	12	14.8%	-0.23 [-0.91, 0.45]
**Total (95% CI)**			**181**			**177**	**100.0%**	**0.09 [-0.17, 0.35]**

Heterogeneity: Chi² = 1.12, df = 2 (P = 0.57); I² = 0%
Test for overall effect: Z = 0.68 (P = 0.50)

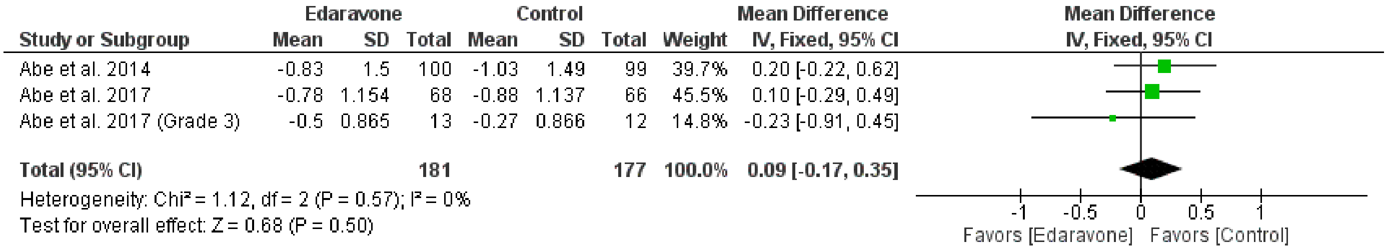

Fig. A4 which demonstrates the pooled mean difference of the Change in pinch strength.

Study or Subgroup	Edaravone Events	Edaravone Total	Control Events	Control Total	Weight	Risk Ratio M-H, Fixed, 95% CI
Abe et al. 2014	7	102	9	104	3.9%	0.79 [0.31, 2.05]
Abe et al. 2017 (Grade 3)	1	13	2	12	0.9%	0.46 [0.05, 4.46]
Brooks et al. 2022	175	318	208	318	91.6%	0.84 [0.74, 0.96]
Eishi-Oskouei et al. 2020	7	10	8	10	3.5%	0.88 [0.53, 1.46]
**Total (95% CI)**		**443**		**444**	**100.0%**	**0.84 [0.74, 0.95]**
Total events	190		227			

Heterogeneity: Chi² = 0.31, df = 3 (P = 0.96); I² = 0%
Test for overall effect: Z = 2.79 (P = 0.005)

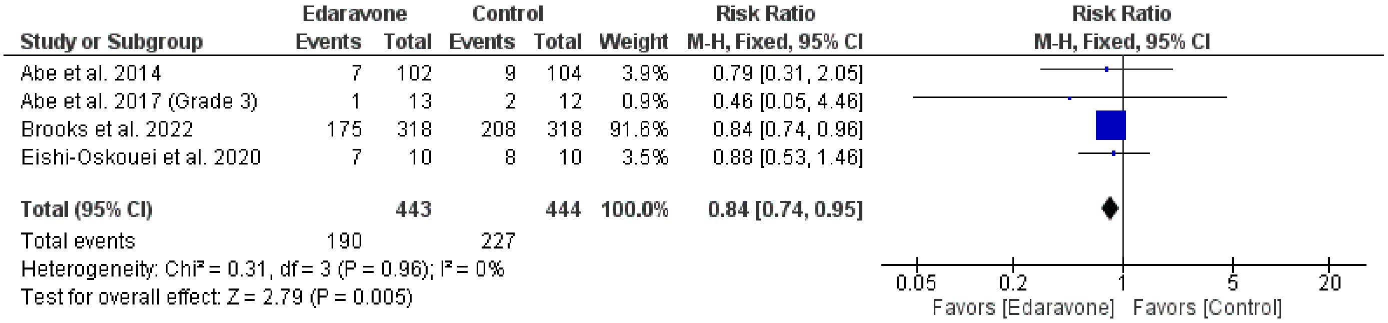

Fig. A5 which demonstrates the pooled risk ratio of musculoskeletal disorders.

Study or Subgroup	Edaravone Events	Edaravone Total	Control Events	Control Total	Weight	Risk Ratio M-H, Fixed, 95% CI
Abe et al. 2014	20	102	16	104	8.7%	1.27 [0.70, 2.32]
Abe et al. 2017 (Grade 3)	4	13	1	12	0.6%	3.69 [0.48, 28.57]
Brooks et al. 2022	133	318	161	318	88.0%	0.83 [0.70, 0.98]
Eishi-Oskouei et al. 2020	3	10	5	10	2.7%	0.60 [0.19, 1.86]
**Total (95% CI)**		**443**		**444**	**100.0%**	**0.88 [0.74, 1.03]**
Total events	160		183			

Heterogeneity: Chi² = 4.29, df = 3 (P = 0.23); I² = 30%
Test for overall effect: Z = 1.62 (P = 0.11)

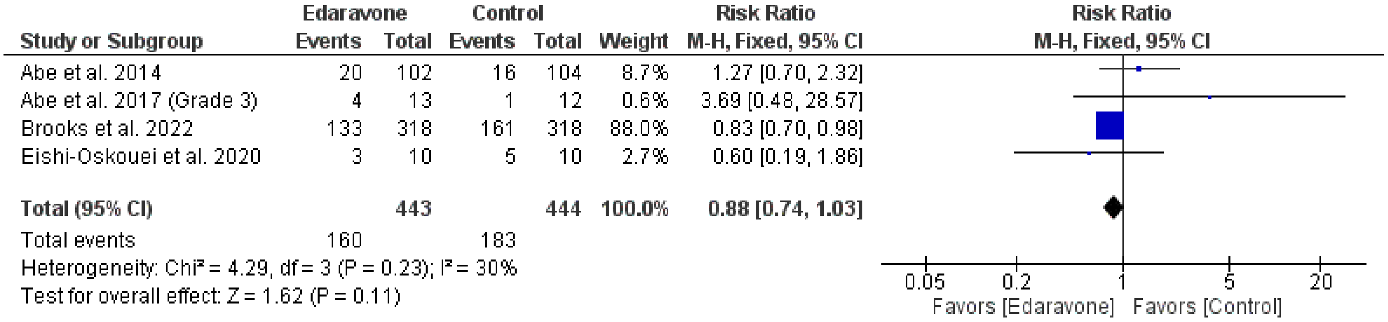

Fig. A6 which demonstrates the pooled risk ratio of gait disturbance.

Study or Subgroup	Edaravone Events	Edaravone Total	Control Events	Control Total	Weight	Risk Ratio M-H, Fixed, 95% CI
Abe et al. 2014	8	102	12	104	7.4%	0.68 [0.29, 1.59]
Abe et al. 2017	8	69	10	68	6.3%	0.79 [0.33, 1.88]
Brooks et al. 2022	134	318	136	318	85.0%	0.99 [0.82, 1.18]
Eishi-Oskouei et al. 2020	4	10	2	10	1.3%	2.00 [0.47, 8.56]
**Total (95% CI)**		**499**		**500**	**100.0%**	**0.96 [0.81, 1.15]**
Total events	154		160			

Heterogeneity: Chi² = 1.88, df = 3 (P = 0.60); I² = 0%
Test for overall effect: Z = 0.43 (P = 0.67)

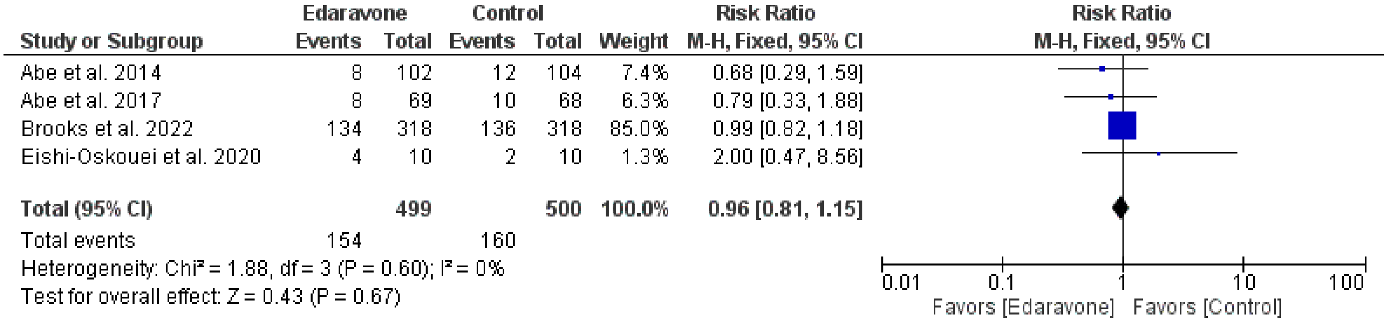

Fig. A7 which demonstrates the pooled risk ratio of dysphagia.

Study or Subgroup	Edaravone Events	Edaravone Total	Control Events	Control Total	Weight	Risk Ratio M-H, Fixed, 95% CI
Abe et al. 2014	13	102	17	104	66.3%	0.78 [0.40, 1.52]
Abe et al. 2017	8	69	8	68	31.7%	0.99 [0.39, 2.48]
Eishi-Oskouei et al. 2020	3	10	0	10	2.0%	7.00 [0.41, 120.16]
**Total (95% CI)**		**181**		**182**	**100.0%**	**0.97 [0.58, 1.62]**
Total events	24		25			

Heterogeneity: Chi² = 2.26, df = 2 (P = 0.32); I² = 12%
Test for overall effect: Z = 0.13 (P = 0.90)

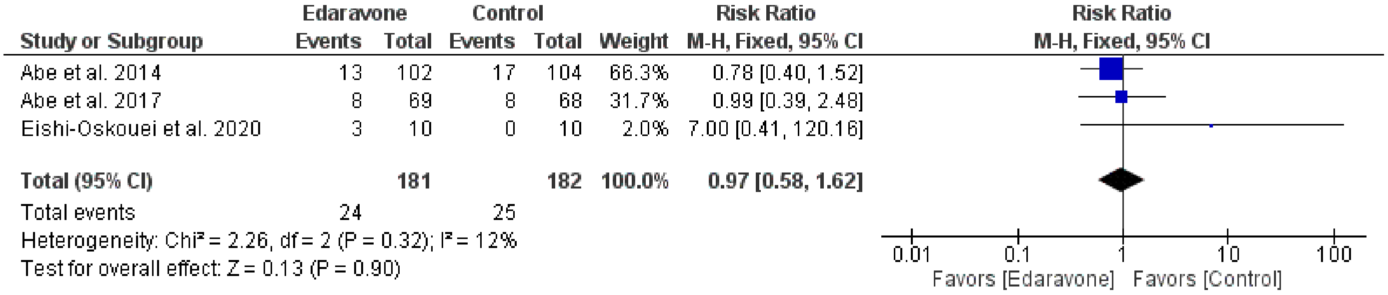

Fig. A8 which demonstrates the pooled risk ratio of constipation.

Study or Subgroup	Edaravone Events	Edaravone Total	Control Events	Control Total	Weight	Risk Ratio M-H, Fixed, 95% CI
Abe et al. 2014	9	102	10	104	71.1%	0.92 [0.39, 2.16]
Abe et al. 2017	5	69	4	68	28.9%	1.23 [0.35, 4.39]
**Total (95% CI)**		**171**		**172**	**100.0%**	**1.01 [0.50, 2.05]**
Total events	14		14			

Heterogeneity: $Chi^2 = 0.14$, df = 1 (P = 0.71); $I^2 = 0\%$
Test for overall effect: Z = 0.02 (P = 0.98)

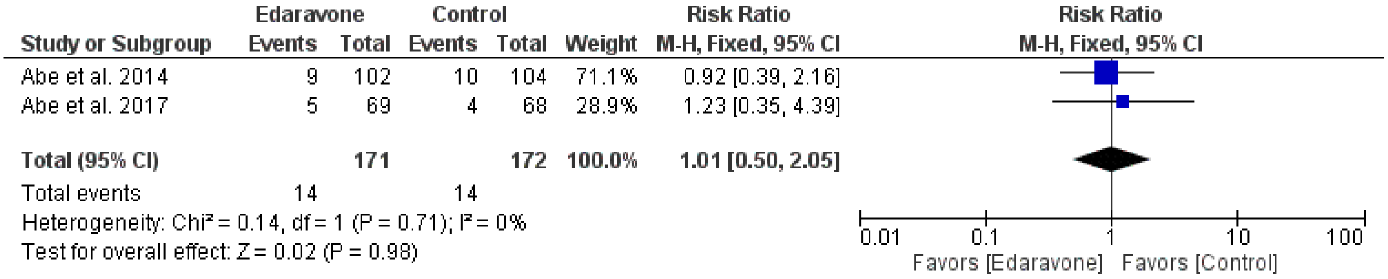

Fig. A9 which demonstrates the pooled risk ratio of insomnia.

Study or Subgroup	Edaravone Events	Edaravone Total	Control Events	Control Total	Weight	Risk Ratio M-H, Fixed, 95% CI
Abe et al. 2017	5	69	2	68	66.0%	2.46 [0.49, 12.27]
Abe et al. 2017 (Grade 3)	3	13	1	12	34.0%	2.77 [0.33, 23.14]
**Total (95% CI)**		**82**		**80**	**100.0%**	**2.57 [0.71, 9.24]**
Total events	8		3			

Heterogeneity: $Chi^2 = 0.01$, df = 1 (P = 0.93); $I^2 = 0\%$
Test for overall effect: Z = 1.44 (P = 0.15)

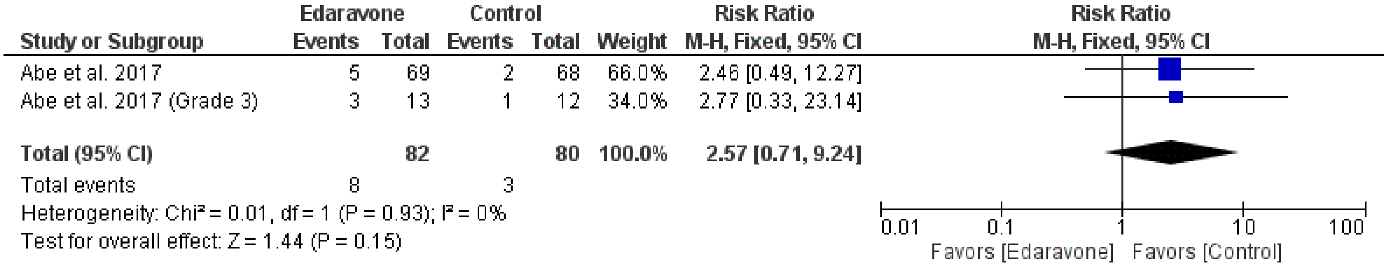

Fig. A10 which demonstrates the pooled risk ratio of upper respiratory tract inflammation.

Study or Subgroup	Edaravone Events	Edaravone Total	Control Events	Control Total	Weight	Risk Ratio M-H, Random, 95% CI
**1.1.1 At six months**						
Abe et al. 2017	68	68	66	66	5.2%	1.00 [0.97, 1.03]
Abe et al. 2017 (Grade 3)	12	13	13	13	3.4%	0.93 [0.75, 1.14]
Brooks et al. 2022	306	318	265	318	5.1%	1.15 [1.09, 1.22]
Houzen et al. 2021	22	22	23	23	4.8%	1.00 [0.92, 1.09]
Lunetta et al. 2020	197	197	290	290	5.3%	1.00 [0.99, 1.01]
Vu et al. 2020	198	223	568	669	5.1%	1.05 [0.99, 1.11]
Witzel et al. 2022	129	130	117	130	5.0%	1.10 [1.04, 1.17]
**Subtotal (95% CI)**		**971**		**1509**	**33.8%**	**1.04 [0.94, 1.14]**
Total events	932		1342			
Heterogeneity: Tau² = 0.01; Chi² = 242.29, df = 6 (P < 0.00001); I² = 98%						
Test for overall effect: Z = 0.75 (P = 0.45)						
**1.1.2 At 12 months**						
Brooks et al. 2022	265	318	221	318	4.8%	1.20 [1.10, 1.31]
Houzen et al. 2021	21	22	19	23	3.4%	1.16 [0.94, 1.42]
Lunetta et al. 2020	195	197	284	290	5.2%	1.01 [0.99, 1.03]
Okada et al. 2018	27	27	29	30	4.7%	1.03 [0.94, 1.13]
Witzel et al. 2022	117	130	108	130	4.7%	1.08 [0.98, 1.19]
**Subtotal (95% CI)**		**694**		**791**	**22.8%**	**1.09 [0.95, 1.25]**
Total events	625		661			
Heterogeneity: Tau² = 0.02; Chi² = 60.58, df = 4 (P < 0.00001); I² = 93%						
Test for overall effect: Z = 1.21 (P = 0.23)						
**1.1.3 At 18 months**						
Brooks et al. 2022	224	318	188	318	4.5%	1.19 [1.06, 1.34]
Houzen et al. 2021	21	22	14	23	0.0%	1.57 [1.12, 2.20]
Lunetta et al. 2020	184	197	255	290	5.1%	1.06 [1.00, 1.12]
Okada et al. 2018	27	27	25	30	3.8%	1.19 [1.01, 1.42]
Witzel et al. 2022	98	130	98	130	4.2%	1.00 [0.87, 1.15]
**Subtotal (95% CI)**		**672**		**768**	**17.5%**	**1.10 [1.01, 1.19]**
Total events	533		566			
Heterogeneity: Tau² = 0.00; Chi² = 6.19, df = 3 (P = 0.10); I² = 52%						
Test for overall effect: Z = 2.30 (P = 0.02)						
**1.1.5 At 24 months**						
Brooks et al. 2022	190	318	156	318	4.1%	1.22 [1.05, 1.41]
Houzen et al. 2021	19	22	14	23	1.9%	1.42 [0.98, 2.05]
Lunetta et al. 2020	166	197	236	290	0.0%	1.04 [0.95, 1.12]
Okada et al. 2018	24	27	17	30	2.1%	1.57 [1.12, 2.20]
Witzel et al. 2022	95	130	76	130	3.7%	1.25 [1.05, 1.49]
**Subtotal (95% CI)**		**497**		**501**	**11.8%**	**1.27 [1.15, 1.41]**
Total events	328		263			
Heterogeneity: Tau² = 0.00; Chi² = 2.22, df = 3 (P = 0.53); I² = 0%						
Test for overall effect: Z = 4.61 (P < 0.00001)						
**1.1.6 At 30 months**						
Brooks et al. 2022	159	318	136	318	3.8%	1.17 [0.99, 1.38]
Houzen et al. 2021	16	23	8	22	0.9%	1.91 [1.03, 3.54]
Lunetta et al. 2020	147	197	208	290	4.6%	1.04 [0.93, 1.16]
Okada et al. 2018	21	27	15	30	1.6%	1.56 [1.03, 2.35]
Witzel et al. 2022	72	130	64	130	3.1%	1.13 [0.89, 1.42]
**Subtotal (95% CI)**		**695**		**790**	**14.0%**	**1.17 [1.01, 1.34]**
Total events	415		431			
Heterogeneity: Tau² = 0.01; Chi² = 7.63, df = 4 (P = 0.11); I² = 48%						
Test for overall effect: Z = 2.16 (P = 0.03)						
**Total (95% CI)**		**3529**		**4359**	**100.0%**	**1.11 [1.04, 1.18]**
Total events	2833		3263			
Heterogeneity: Tau² = 0.02; Chi² = 715.79, df = 24 (P < 0.00001); I² = 97%						
Test for overall effect: Z = 3.27 (P = 0.001)						
Test for subgroup differences: Chi² = 9.16, df = 4 (P = 0.06), I² = 56.3%						

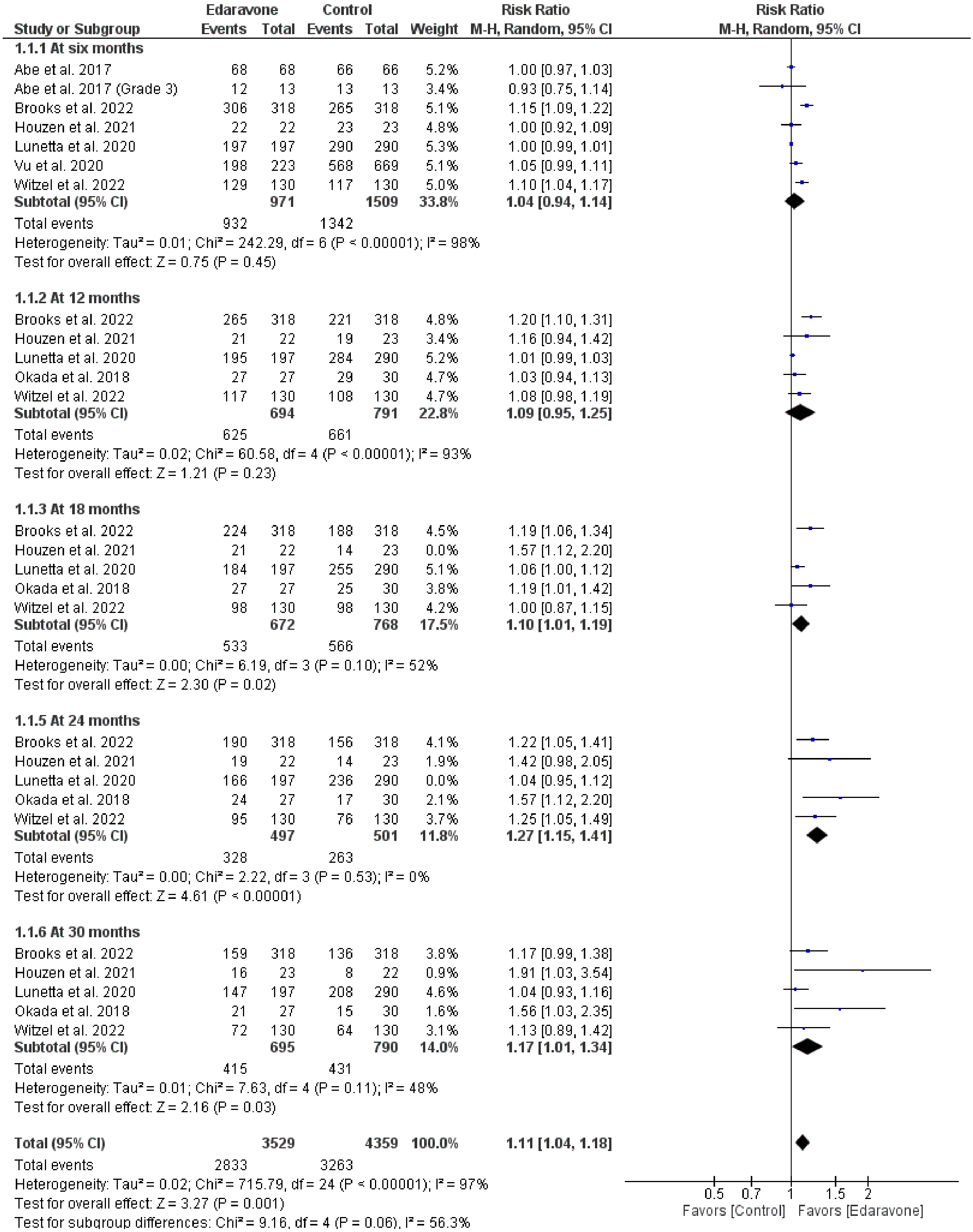

Fig. A11 which demonstrates the pooled risk ratio of Survival rates.

Study or Subgroup	Edaravone Mean	Edaravone SD	Edaravone Total	Control Mean	Control SD	Control Total	Weight	Mean Difference IV, Random, 95% CI
**2.2.1 After one cycle**								
Abe et al. 2017	-0.95	3.78	69	-0.91	3.656	67	6.9%	-0.04 [-1.29, 1.21]
Abe et al. 2017 (Grade 3)	-0.24	7.72	13	-0.72	5.038	12	3.8%	0.48 [-4.59, 5.55]
Witzel et al. 2022	-0.97	1.1	62	-1	1.284	30	7.2%	0.03 [-0.50, 0.56]
**Subtotal (95% CI)**			**144**			**109**	**17.9%**	**0.02 [-0.47, 0.51]**
Heterogeneity: Tau² = 0.00; Chi² = 0.04, df = 2 (P = 0.98); I² = 0%								
Test for overall effect: Z = 0.09 (P = 0.93)								
**2.2.2 After three cycles**								
Abe et al. 2017	-2.265	4.72	68	-2.725	4.135	65	6.7%	0.46 [-1.05, 1.97]
Abe et al. 2017 (Grade 3)	-1.79	7.614	13	-2.73	5.94	12	3.6%	0.94 [-4.39, 6.27]
Eishi-Oskouei et al. 2020	-3.6	7.923	10	-7.2	7.549	10	2.8%	3.60 [-3.18, 10.38]
Fortuna et al. 2019	-2.41	7.193	31	-2.07	3.849	50	5.7%	-0.34 [-3.09, 2.41]
Lunetta et al. 2020	-3.67	4.1	197	-2	2.98	290	7.1%	-1.67 [-2.34, -1.00]
Vu et al. 2020	-3	3.73	223	-8.67	7.429	669	7.1%	5.67 [4.92, 6.42]
**Subtotal (95% CI)**			**542**			**1096**	**33.1%**	**1.34 [-2.38, 5.07]**
Heterogeneity: Tau² = 18.74; Chi² = 210.57, df = 5 (P < 0.00001); I² = 98%								
Test for overall effect: Z = 0.71 (P = 0.48)								
**2.2.3 After six cycles**								
Abe et al. 2014	-5.7	8.5	100	-6.35	8.375	99	6.1%	0.65 [-1.69, 2.99]
Abe et al. 2017	-4.506	5.839	68	-6.786	6.01	61	6.3%	2.28 [0.23, 4.33]
Abe et al. 2017 (Grade 3)	-2.882	7.25	9	-5.28	7.128	12	3.1%	2.40 [-3.82, 8.62]
Eishi-Oskouei et al. 2020	-8.4	9.31	10	-13.4	8.504	10	2.3%	5.00 [-2.82, 12.82]
Fortuna et al. 2019	-3.068	7.13	31	-2.82	4.556	50	5.7%	-0.25 [-3.06, 2.56]
Lunetta et al. 2020	-5.33	5.22	122	-5.01	3	199	7.0%	-0.32 [-1.34, 0.70]
Okada et al. 2018	-2.857	3.775	21	-9.387	3.157	26	0.0%	6.53 [4.51, 8.55]
**Subtotal (95% CI)**			**340**			**431**	**30.5%**	**0.63 [-0.55, 1.81]**
Heterogeneity: Tau² = 0.61; Chi² = 7.07, df = 5 (P = 0.22); I² = 29%								
Test for overall effect: Z = 1.04 (P = 0.30)								
**2.2.4 After nine cycles**								
Eishi-Oskouei et al. 2020	-11.5	10.26	10	-16.9	8.91	10	2.1%	5.40 [-3.02, 13.82]
Lunetta et al. 2020	-7.83	6.06	67	-2.83	6.1	51	6.2%	-5.00 [-7.22, -2.78]
**Subtotal (95% CI)**			**77**			**61**	**8.3%**	**-0.63 [-10.69, 9.44]**
Heterogeneity: Tau² = 44.21; Chi² = 5.48, df = 1 (P = 0.02); I² = 82%								
Test for overall effect: Z = 0.12 (P = 0.90)								
**2.2.5 After 12 cycles**								
Eishi-Oskouei et al. 2020	-16	11.25	10	-22.2	8.785	10	1.9%	6.20 [-2.65, 15.05]
Lunetta et al. 2020	-9.67	7.468	44	-8.33	8.19	39	5.2%	-1.34 [-4.73, 2.05]
Okada et al. 2018	-5.714	5.91	13	-10.2	9.59	13	3.1%	4.49 [-1.64, 10.61]
**Subtotal (95% CI)**			**67**			**62**	**10.2%**	**2.06 [-2.87, 6.98]**
Heterogeneity: Tau² = 10.23; Chi² = 4.34, df = 2 (P = 0.11); I² = 54%								
Test for overall effect: Z = 0.82 (P = 0.41)								
**Total (95% CI)**			**1170**			**1759**	**100.0%**	**0.78 [-0.66, 2.22]**
Heterogeneity: Tau² = 7.41; Chi² = 266.24, df = 19 (P < 0.00001); I² = 93%								
Test for overall effect: Z = 1.06 (P = 0.29)								
Test for subgroup differences: Chi² = 1.88, df = 4 (P = 0.76), I² = 0%								

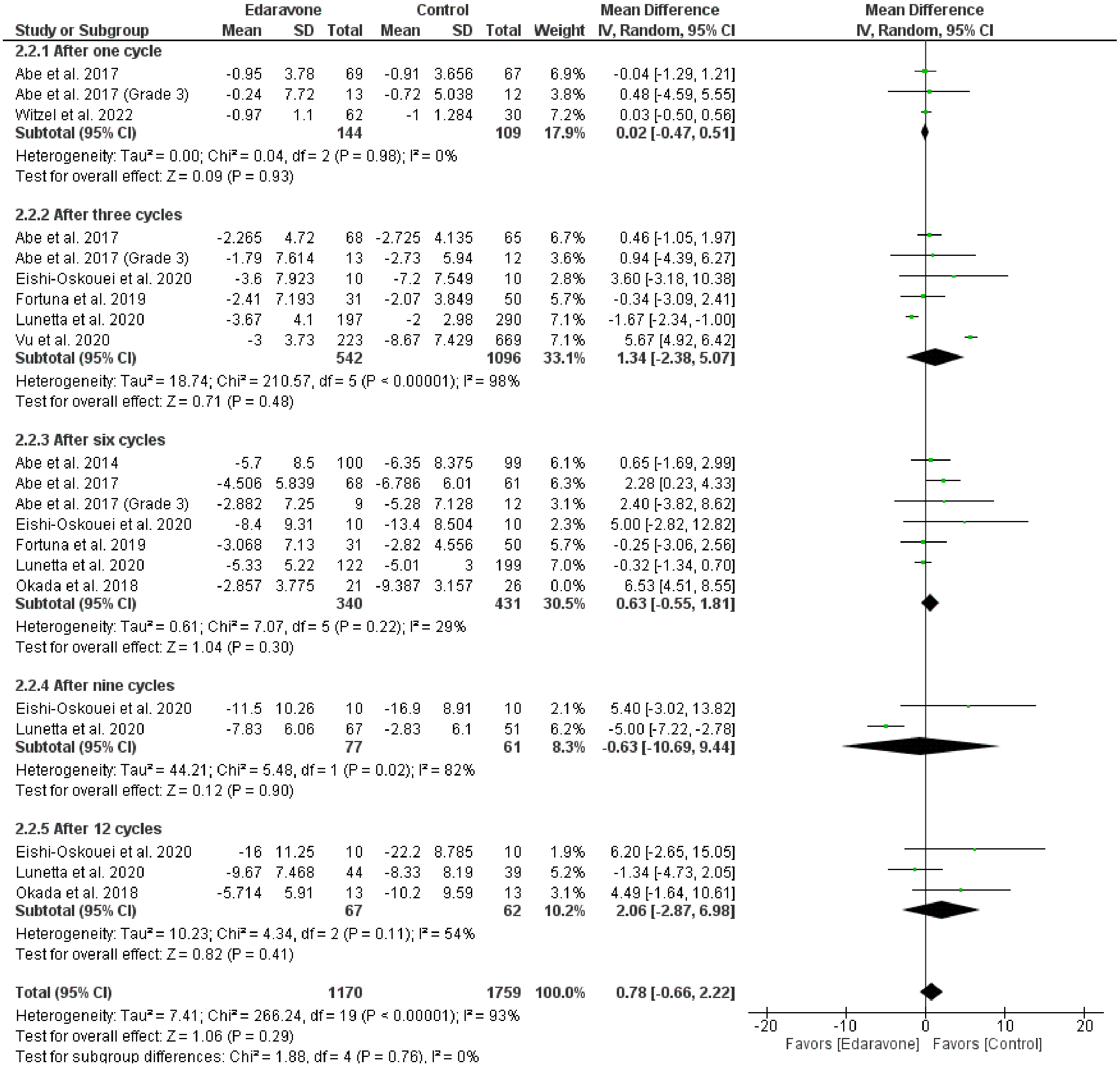

Fig. A12 which demonstrates the pooled mean difference of the Change in ALSFRS-R score after six cycles after sensitivity analysis.

	Edaravone			Control				Mean Difference	Mean Difference
Study or Subgroup	Mean	SD	Total	Mean	SD	Total	Weight	IV, Random, 95% CI	IV, Random, 95% CI
Abe et al. 2014	-14.57	24.1	100	-17.49	23.78	99	33.1%	2.92 [-3.73, 9.57]	
Abe et al. 2017	-15.61	19.87	68	-20.4	20.147	66	31.8%	4.79 [-1.99, 11.57]	
Abe et al. 2017 (Grade 3)	-18.75	16.51	13	-15.69	15.865	12	9.1%	-3.06 [-15.75, 9.63]	
Fortuna et al. 2019	-13.9	15.9	31	-17	18	50	26.0%	3.10 [-4.40, 10.60]	
Lunetta et al. 2020	-16.167	20.99	128	-9.067	12.746	215	0.0%	-7.10 [-11.12, -3.08]	
**Total (95% CI)**			**212**			**227**	**100.0%**	**3.02 [-0.81, 6.84]**	

Heterogeneity: Tau² = 0.00; Chi² = 1.14, df = 3 (P = 0.77); I² = 0%
Test for overall effect: Z = 1.55 (P = 0.12)

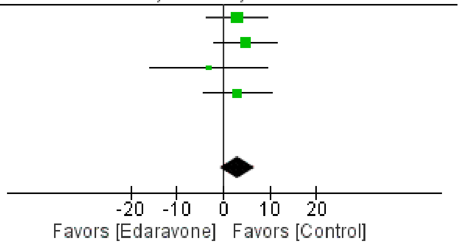

Fig. A13 which demonstrates the pooled mean difference of the Change in FVC after sensitivity analysis.
